# Activation of the TRPML1 Ion Channel Induces Proton Secretion in the Human Gastric Parietal Cell Line HGT-1

**DOI:** 10.3390/ijms25168829

**Published:** 2024-08-13

**Authors:** Alina Ulrike Mueller, Gaby Andersen, Phil Richter, Veronika Somoza

**Affiliations:** 1 TUM School of Life Sciences Weihenstephan, Technical University of Munich, Alte Akademie 8, 85354 Freising, Germany; 2 Leibniz Institute for Food Systems Biology at the Technical University of Munich, Lise-Meitner-Str. 34, 85354 Freising, Germany; 3 Chair of Nutritional Systems Biology, TUM School of Life Sciences, Technical University of Munich, Lise-Meitner-Str. 34, 85354 Freising, Germany; 4 Department of Physiological Chemistry, Faculty of Chemistry, University of Vienna, Josef-Holaubek-Platz 2 (UZA II), 1090 Wien, Austria

**Keywords:** TRPML1, Mucolipin1, MCOLN1, calcium, Lamp1

## Abstract

The lysosomal Ca^2+^ channel TRPML1 was found to be responsible for gastric acid secretion in murine gastric parietal cells by inducing the trafficking of H^+^/K^+^-ATPase containing tubulovesicles to the apical membrane. Therefore, we hypothesized a similar role of TRPML1 in regulating proton secretion in the immortalized human parietal cell line HGT-1. The primary focus was to investigate the involvement of TRPML1 in proton secretion using the known synthetic agonists ML-SA1 and ML-SA5 and the antagonist ML-SI3 and, furthermore, to identify food-derived compounds that target the channel. Proton secretion stimulated by ML-SA1 was reduced by 122.2 ± 22.7% by the antagonist ML-SI3. The steroid hormone 17β-estradiol, present in animal-derived foods, diminished the proton secretory effect of ML-SA1 by 63.4 ± 14.5%. We also demonstrated a reduction in the proton secretory effects of ML-SA1 and ML-SA5 on TRPML1 knock-down cells. The food-derived compounds sulforaphane and trehalose promoted proton secretion in HGT-1 cells but may act independently of TRPML1. Also, histamine- and caffeine-induced proton secretion were affected by neither the TRPML1 antagonist ML-SI3 nor the TRPML1 knock-down. In summary, the results obtained suggest that the activation of TRPML1 promotes proton secretion in HGT-1 cells, but the channel may not participate in canonical signaling pathways.

## 1. Introduction

Elucidating the functions of various chemosensory receptors in non-sensory tissues has been of increasing interest for several years. These include taste and olfactory receptors and transient receptor potential (TRP) channels responsible for chemesthetic perceptions in the oral cavity. The TRP ion channel family comprises integral membrane proteins that function as ion channels, and they are classified into seven subfamilies: TRPC (canonical), TRPV (vanilloid), TRPM (melastatin), TRPP (polycystin), TRPML (mucolipin), TRPA (ankyrin), and TRPN (NOMPC-like) [1]. TRP ion channels are present in numerous tissues and cell types, where they play a role in diverse physiological processes, including ion homeostasis, motile functions, and sensing various stimuli [1].

**Figure 1:**
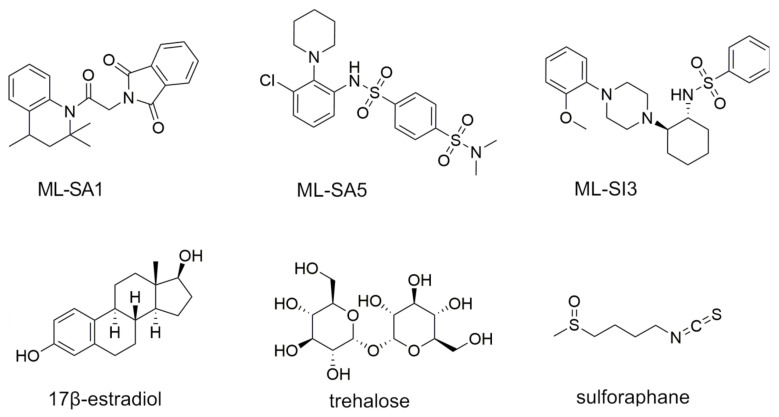
Chemical structures of the synthetic TRPML1 agonists ML-SA1 and ML-SA5, the antagonist ML-SI3, and the potential food-derived TRPML1 activity modulators sulforaphane, trehalose, and 17β-estradiol.

The TRP channel TRPML1 belongs to the mucolipin TRP channel family, which consists of three distinct members: TRPML1, TRPML2, and TRPML3. The human TRPML1 channel, comprising 580 amino acids with a molecular mass of 65 kDa, is mainly located in the membrane of late endosomes and lysosomes responsible for membrane trafficking and autophagy-related processes [2]. As a cation channel, it is permeable to Ca^2+^, Fe^2+^, Zn^2+^, Na^+^, and K^+^ and regulates their transport to the cytosol [2]. Pharmacokinetic studies demonstrated that TRPML1 interacts with various structurally barely related compounds. For example, the channel is activated by phosphatidylinositol 3,5-bisphosphate (PI(3,5)P_2_) [3,4], rapamycin [5], and the synthetic agonists ML-SA1 [6] and ML-SA5 [7]. Described inhibitors include VacA, a toxin produced by *Heliobacter pylori* [8]; phosphatidylinositol 4,5-bisphosphate (PI(4,5)P_2_) [3]; and the synthetic antagonist ML-SI3 [9]. Furthermore, it was shown that TRPML1 activation is potentiated at lower surrounding pH values [9,10]. TRPML1 has also been described as a proton leak channel in lysosomes that prevents these organelles from becoming too acidic [11]. Regarding food-derived compounds, sulforaphane, an isothiocyanate formed from glucoraphanin in cruciferous vegetables after slicing, with high concentrations in broccoli of 48.7 mg/100 g, was shown to stimulate TRPML1 activity [12,13]. Li and colleagues [12] studied the effects of sulforaphane on autophagy and lysosomal functions at concentrations of 5 µM–10 µM in HeLa cells and suggested that a mild elevation in intracellular reactive oxygen species by sulforaphane enhances the release of Ca^2+^ through TRPML1 channels and other nonidentified Ca^2+^ channels. Also, the disaccharide trehalose might modulate TRPML1 activity, since it was proposed that trehalose acts on lysosomes by inducing a transient lysosomal membrane permeability, causing a Ca^2+^ efflux at a concentration of 100 mM [14]. In addition, trehalose was found to activate TRPML1 expression in models of motoneurons and activates TRPML1 by increasing PI(3,5)P_2_ levels [14,15]. On the other hand, 17β-estradiol, a steroid hormone from animal-derived foods, was found to directly inhibit TRPML1 [16]. Chemical structures of the well-described synthetic TRPML1 agonists ML-SA1 (at pH_lysosomal_ EC_50_ = 9.7 µM; at pH_7.4_ EC_50_ = 15.3 µM [9]) and ML-SA5 (EC_50_ = 0.885 µM [17]), the antagonist ML-SI3 (IC_50_ = 4.7 µM [16]), and the potential TRPML1 activity modulators sulforaphane (no EC_50_ data available), trehalose (no EC_50_ data available), and 17β-estradiol (IC_50_ = 5.3 µM [16]) are shown in Figure 1.

Mutations in the *MCOLN1* gene encoding for TRPML1 are responsible for mucolipidosis type IV, which is an autosomal-recessive lysosomal storage disorder characterized by severe psychomotor delay, progressive visual impairment, and an additional stomach parietal cell phenotype of selective vacuolation and constitutive achlorhydria [18]. This medical condition indicates a critical role of TRPML1 in human gastric acid secretion. The participation of TRPML1 in gastric acid secretion was already investigated in murine models [7,19]. TRPML1-knockout mice showed impaired histamine-induced gastric acid secretion and reduced H^+^/K^+^-ATPase expression, resulting in hypochlorhydria and hypergastrinemia [18,19]. By contrast, the transgenic overexpression of TRPML1 in mouse parietal cells resulted in constitutive acid secretion [7]. Thus, the TRPML1 channel was identified as a histamine-activated Ca^2+^ channel in lysosomes and tubulovesicles (TVs) necessary for gastric acid secretion in mice [7,19]. It was proposed that TRPML1 is required for TV formation and for trafficking TVs containing H^+^/K^+^-ATPases to the apical membrane [7].

A well-established model for analyzing the mechanisms of human gastric acid secretion in vitro is the HGT-1 cell line [20,21,22,23,24]. HGT-1 cells express histamine H_2_ receptors, mediating one of the main pathways leading to proton secretion. The binding of histamine to its receptor leads to adenylyl cyclase activation and cAMP production, thereby initializing the trafficking and exocytosis of H^+^/K^+^-ATPase-enriched tubulovesicles toward the apical membrane [25,26]. Furthermore, HGT-1 cells express all principal receptors and transporters involved in proton secretion [22,27]. Moreover, HGT-1 cells have been established as a suitable cell model for investigating the effects of various food-derived compounds on chemoreceptor-mediated proton secretion, ranging from bitter-tasting peptides and amino acids to noncaloric sweeteners and small molecules like the bitter compound caffeine [20,21,22,28]. Especially the bitter taste-like signaling pathway via the G-protein-coupled receptors TAS2Rs has been intensively investigated in HGT-1 cells and shown to be highly comparable to taste cells [21,22,28]. Recently, also the TRPM4 and TRPM5 channels were found to be involved in TAS2R-activated proton secretion in HGT-1 cells via Na^+^ influx induced by increased cytosolic Ca^2+^ concentrations [21]. However, the exact underlying signaling pathways have not been fully elucidated yet.

Based on the previous findings from mouse parietal cells, we hypothesized the TRPML1 channel to be an essential component of proton secretion in cultured human gastric parietal cells, irrespective of the triggering compound and the signaling pathway induced by it. To verify our hypothesis, we studied the participation of TRPML1 in proton secretion as part of the paracrine signaling pathway induced by histamine acting via cAMP and the taste-like signaling pathway induced by the bitter compound caffeine acting via Ca^2+^ mobilization from the ER. Furthermore, we applied the synthetic small molecules ML-SA1, ML-SA5, and ML-SI3 as direct modulators of TRPML1. The described TRPML1-targeting food-derived compounds 17β-estradiol, sulforaphane, and trehalose were also chosen to investigate TRPML1’s general involvement in proton secretion in the human parietal cell line HGT-1.

## 2. Results

### 2.1. TRPML1 Is Expressed in the Parietal Cell Line HGT-1

**Figure 2:**
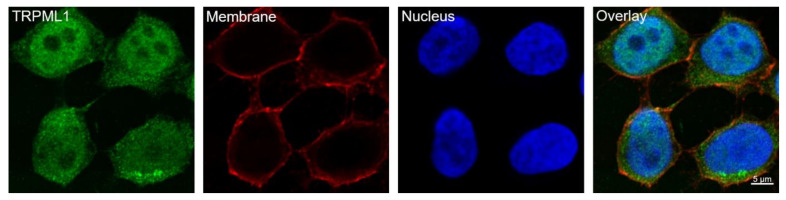
TRPML1 is expressed in HGT-1 cells detected with super-resolution imaging. Fluorescent-labeled TRPML1 channels with anti-TRPML1 antibody and anti-Rabbit IgG with Alexa Fluor 488 fluorophore (green) are localized in the cell membrane, cytoplasm, and nucleus, in contrast to membrane staining with ConA and streptavidin-Alexa Fluor 633 (red) and nucleus staining with Hoechst-33342 (blue). The Zeiss LSM 780 microscope (Carl Zeiss AG, Munich, Germany) with airyscan detection (Carl Zeiss AG, Munich, Germany) and a 40×/1.2 Imm Korr DIC M27 objective lens (Carl Zeiss AG, Munich, Germany) was used for image acquisition. Scale bar: 5 μm.

Since TRPML1 has recently been detected at the mRNA level in HGT-1 cells [21], we first investigated its protein expression by immunocytochemistry. Specific antibody binding could be verified after staining with the anti-TRPML1 antibody and the corresponding blocking peptide (Figure S1). A high-resolution image of the immunostained HGT-1 cell visualizes the TRPML1 fluorescence signal in the plasma membrane, cytoplasm, and nucleus (Figure 2).

### 2.2. TRPML1 Activation Induces Proton Secretion in HGT-1 Cells

**Figure 3:**
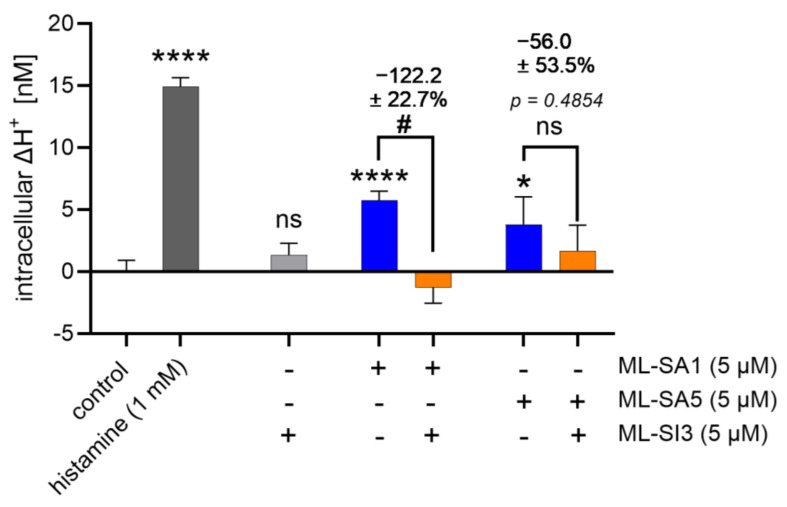
Effect on proton secretion in HGT-1 cells incubated with 5 µM of the synthetic TRPML1 agonists ML-SA1 and ML-SA5 and the antagonist ML-SI3. Intracellular ΔH^+^ concentrations in nM are shown as mean ± SEM, *n* = 4–5, t. r. = 4–6. Statistics: Student’s *t*-test of untreated compared to treated cells (two-tailed, unpaired); significant differences are indicated with ns = not significant, * = *p* ≤ 0.05, and **** = *p* ≤ 0.0001. Student’s *t*-test of activator-only treated cells compared to cells treated with the co-incubation of ML-SI3 with ML-SA1 or ML-SA5 (two-tailed, unpaired); significant differences are indicated with ns = not significant; # = *p* ≤ 0.05.

According to our hypothesis, applying the synthetic TRPML1 agonists ML-SA1 and ML-SA5 and the antagonist ML-SI3 would modulate proton-secreting activity in HGT-1 cells, as determined using a well-established proton secretion assay [20,22,23,29]. With pH-sensitive dyes such as SNARF-AM, it is possible to measure intracellular pH values and thereby determine intracellular H^+^ concentrations. This allows for the calculation of alterations in intracellular H^+^ concentrations (ΔH^+^) upon the treatment of the cells by subtracting the intracellular proton concentrations of treated cells from the intracellular proton concentrations of untreated cells. Therefore, the ΔH^+^ describes the extent of the induced proton secretion. We could show that both agonists, ML-SA1 and ML-SA5, are generally sufficient to induce proton secretion in HGT-1 cells, with ΔH^+^ values of 5.75 ± 0.7 nM and 3.81 ± 1.1 nM, respectively (Figure 3). To classify the effect size of proton secretion induced by TRPML1 activation, we applied 1 mM histamine, which has an EC_50_ value of 50 µM regarding adenylyl cyclase activity in human gastric mucosa but a saturating concentration of 1 mM [30]. In comparison, proton secretion induced by TRPML1 activation is around 39% of the proton secretion induced by 1 mM histamine (ΔH^+^: 14.9 ± 0.7 nM, *p* ≤ 0.0001) (Figure 3). The TRPML1 antagonist ML-SI3 expectedly had no effect on proton secretion at a concentration of 5 µM but reduced the proton secretion induced by 5 µM ML-SA1 by 122.2 ± 22.7% (*p* ≤ 0.05). This reduction of above 100% was probably due to the fact that the intracellular pH of unstimulated cells is about 7.6, and proton secretion leads to an increase in the intracellular pH as more protons are secreted. However, if the secretion is inhibited, the pH value can shift below pH 7.6 because more protons are present in the cell than are secreted, which is why it is possible to obtain a negative intracellular ΔH^+^ value and, therefore, also a reduction above 100%. On the contrary, ML-SI3 could not reduce the proton secretory effect of 5 µM ML-SA5 (−56.0 ± 53.5%, *p* > 0.05) (Figure 3). Notably, ML-SA1 and ML-SA5 concentrations higher than 5 µM did not increase proton secretion (Figure S2). Also, Rühl et al. used 5 µM ML-SA1 to activate TRPML1 [16]. Therefore, a concentration of 5 µM ML-SA1, ML-SA5, and ML-SI3 was chosen for the following experiments.

### 2.3. TRPML1 Activation Leads to Increased Cytosolic Ca^2+^ Concentrations

**Figure 4:**
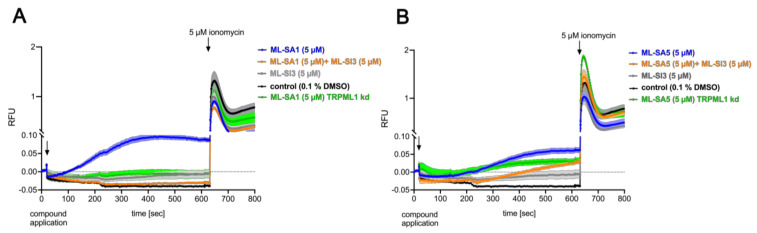
Inhibitory effect of ML-SI3 on ML-SA1- and ML-SA5-stimulated HGT-1 cells in the calcium mobilization assay. (**A**): Co-incubation of ML-SA1 with ML-SI3 in the calcium mobilization assay. The ML-SA1 signal in TRPML1 kd cells is shown in green. (**B**): Co-incubation of ML-SA5 with ML-SI3 in the calcium mobilization assay. The ML-SA5 signal in TRPML1 kd cells is shown in green. A 5 µM ionomycin was applied as a positive control. Data are shown as relative fluorescence (RFU) normalized to baseline fluorescence *n* = 3, t. r. = 2.

Since activation of TRPML1 in parietal cells from mice led to the mobilization of Ca^2+^ from the TVs into the cytosol [7], we next analyzed whether TRPML1 activation generally increases cytosolic Ca^2+^ levels in HGT-1 cells. The applications of the agonists ML-SA1 (5 µM, *p* ≤ 0.0001) and ML-SA5 (5 µM, *p* ≤ 0.0001) both led to an increase in the cytosolic Ca^2+^ concentration (Figure 4). The ML-SA1-induced effect was fully inhibited by the antagonist ML-SI3 (*p* ≤ 0.0001) (Figure 4A). In contrast to its effect on ML-SA5-induced proton secretion, ML-SI3 could reduce the Ca^2+^ signal of ML-SA5 (*p* ≤ 0.001), but only by around 35% compared to the control (Figure 4B). The application of ML-SA1 and ML-SA5 in nominal Ca^2+^-free buffer abolished the signal completely (Figure S3), indicating a Ca^2+^ influx rather than a Ca^2+^ release from intracellular stores. A TRPML1-specific knock-down (kd) neutralized the ML-SA1-induced increase in cytosolic Ca^2+^ concentration completely and the ML-SA5-induced effect by 35%. The comparison of the Ca^2+^ signals induced by ML-SA1 and ML-SA5 to histamine, however, is problematic since histamine evoked a sharp increase after application, possibly associated with artifacts (Figure S4).

### 2.4. H^+^/K^+^-ATPase Accumulation and Vacuolar Apical Compartment Formation in HGT-1 Cells

**Figure 5:**
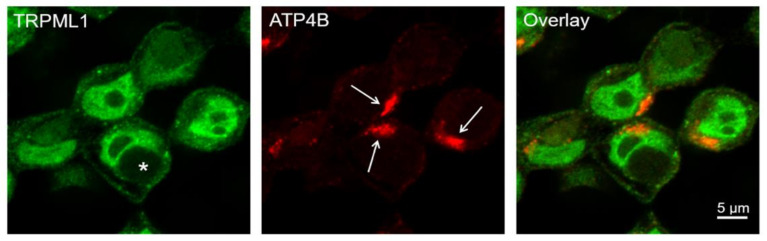
Super-resolution image of 5 µM ML-SA1-incubated HGT-1 cells stained for TRPML1 (green) and the β-subunit of H^+^/K^+^-ATPase ATP4B (red) obtained with an airyscan detector: For image acquisition, a Zeiss LSM 780 microscope equipped with airyscan detection and a 40×/1.2 Imm Korr DIC M27 objective lens was used. Airyscan image processing was performed using the ZEN 2.3 SP1 black program. The scale bar represents 5 μm. The H^+^/K^+^-ATPase accumulation is indicated in the direction of the arrow. The vacuolar apical compartment formation is displayed with the star.

**Figure 6:**
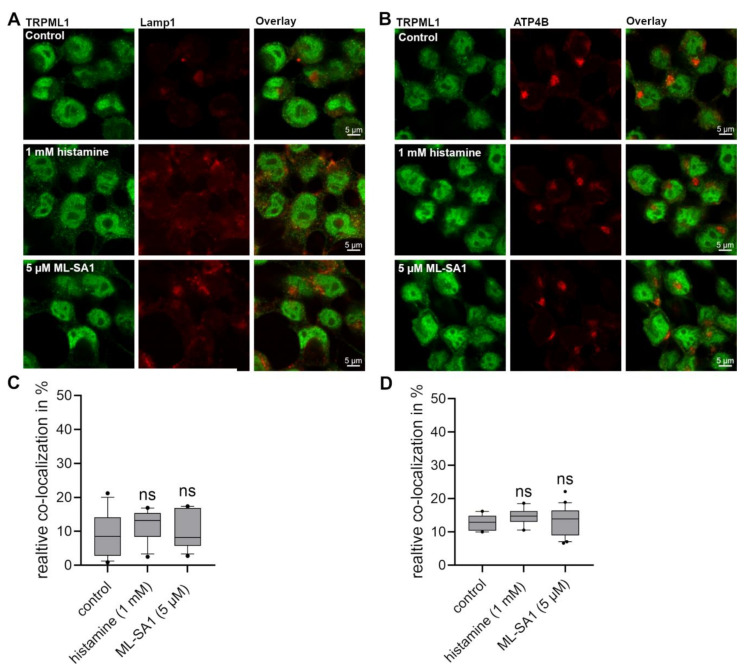
Co-localization of TRPML1 channels Lamp-1 and β-subunit of H^+^/K^+^-ATPase (ATP4B) in HGT-1 cells. Cells were incubated with either 1 mM histamine or 5 µM ML-SA1 for 10 min before staining with anti-TRPML1 antibody (green) and anti-human-CD107a (**A** in red) for Lamp-1. The β-subunit of H^+^/K^+^-ATPase was stained with anti-ATP4B antibody (**B** in red). Scale bar represents 5 μm. The co-localization was calculated for TRPML1 with Lamp-1 (**C**) and ATP4B (**D**) based on the pixel count. Data is presented as box plots with 10th and 90th percentiles. Data points outside of this range are indicated as dots, *n* = 1–2, t. r. = 10–20. Statistics: Student’s *t*-test of control compared treated cells (two-tailed, paired); ns: not significant.

Upon the stimulation of parietal cells, apical canalicular membranes are engulfed by the cell, forming multiple actin-wrapped vacuoles known as vacuolar apical compartments [31]. Previous work showed that ML-SA5 induced vacuolar apical compartment formation in the parietal cells of mice [7]. As shown in Figure 5, we also demonstrated vacuolar apical compartment formation in HGT-1 cells upon the activation of TRPML1 with ML-SA1 (untreated control is shown in Figure 6B).

The main fraction of H^+^/K^+^-ATPases in resting parietal cells has been demonstrated to be localized in tubulovesicles and mobilized to the cell surface upon stimulation [25]. In this work, we could detect the β-subunit of H^+^/K^+^-ATPase accumulated only on one side of the cell, which may indicate the apical membrane (Figure 5 and Figure 6B).

Fragmented phalloidin (F-actin) staining is considered to demonstrate the resting state of the cell and continuous phalloidin staining to demonstrate the stimulated state of the cell [7,32]. In our experiments, however, stimulated cells showed no different phalloidin staining from unstimulated cells, supporting the ready-to-release proton state of HGT-1 cells (untreated and 1 mM histamine-treated cells show exemplarily the stimulated state, as depicted in Figure S5).

### 2.5. Stimulation with Histamine and ML-SA1 Causes the Shifting of Lamp-1 to the Cell Membrane in Live-Cell Staining in HGT-1 Cells

By studying constitutively active TRPML1 channels, Dong et al. [33] hypothesized that active TRPML1 channels trigger intra-lysosomal Ca^2+^ release and, consequently, the appearance of TRPML1 and the lysosomal protein Lamp-1 at the plasma membrane. Since TRPML1 was identified as a tubulovesicular channel responsible for TV exocytosis for gastric acid secretion in mice, we investigated Lamp-1 expression at the cell membrane after stimulation. Live-cell staining using a flow cytometer was performed to verify whether a shift of Lamp-1-containing lysosomes or TVs occurs after stimulation. The detected mean fluorescence for untreated cells was 27.2 ± 0.40 AU, that for 1 mM histamine-treated cells was 28.4 ± 0.46 AU (*p* = 0.0645), and that for 5 µM ML-SA1-treated cells was 28.5 ± 0.51 AU (*p* = 0.0452). About 5000 cells were analyzed to calculate the mean fluorescence and the SEM; statistical significance was tested with an ordinary one-way ANOVA and Fisher’s LSD test. This might indicate a small increase in Lamp-1 at the cell membrane after stimulation with histamine and ML-SA1.

### 2.6. No Difference in the Co-Localization of TRPML1 with Lamp-1 and H^+^/K^+^-ATPase Could Be Detected in HGT-1 Cells after Exposure to Histamine and ML-SA1

Following the assumption that histamine and ML-SA1 trigger lysosome or TV trafficking, we incubated HGT-1 cells with these compounds, followed by staining for TRPML1, Lamp-1, and ATP4B and analyzing their assumed co-localization via fluorescence microscopy (Figure 6). Lamp-1 was chosen as a marker of lysosomes and ATP4B, the β-subunit of H^+^/K^+^-ATPase, as a marker of TVs. We detected a mean co-localization of TRPML1 with Lamp-1 and H^+^/K^+^-ATPase of 9.1 ± 1.8% and 12.9 ± 0.7%, respectively, with no difference in stimulated and non-stimulated cells quantitated. However, as already demonstrated in murine models, TRPML1 is present in H^+^/K^+^-ATPase-positive TVs and Lamp-1-containing lysosomes [7,19]. In agreement with the co-immunoprecipitation experiments with TRPML1 and H^+^/K^+^-ATPase of Chandra et al. [19], but in contrast to Sahoo et al. [7], we could not detect a high co-localization of TRPML1 with the H^+^/K^+^-ATPase β-subunit of 60–80% and thereby strengthen the assumption that TRPML1 in parietal cells is more prominently associated with TVs as compared to lysosomes. However, we cannot rule out that TVs sequester only the α-subunit of the H^+^/K^+^-ATPase to the apical membrane.

### 2.7. The Potential TRPML1 Activity Modulators Sulforaphane, Trehalose, and 17β-Estradiol Impact Proton Secretion in HGT-1 Cells

**Figure 7:**
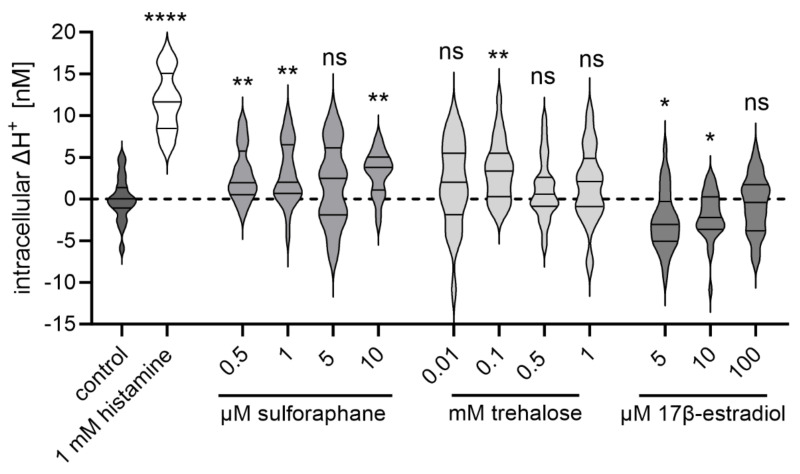
Proton secretion-inducing effect of sulforaphane, trehalose, and 17β-estradiol on HGT-1 cells. Intracellular ΔH^+^ concentrations in nM are shown as a violin plot, *n* = 4–5, t. r. = 4–6. Statistics: Student’s *t*-test (two-tailed, unpaired); significant differences are indicated with ns = not significant; * = *p* ≤ 0.05; ** = *p* ≤ 0.01; **** = *p* ≤ 0.0001.

Since food and food-derived compounds are initiators of proton secretion, we next analyzed whether not only synthetic ligands of TRPML1 but also food-derived compounds that were associated with TRPML1 activity modulation and the regulation of lysosomal biogenesis and autophagy via TRPML1 can impact the proton secretion in HGT-1 cells. Therefore, the TRPML1 activity modulators sulforaphane [12], trehalose [14,15], and 17β-estradiol [16] in the concentration ranges described were chosen based on previous reports. In the demonstrated experiments, sulforaphane (*p* ≤ 0.01) and trehalose (*p* ≤ 0.01) stimulated the secretion of protons in HGT-1 cells (Figure 7). In contrast, 17β-estradiol inhibited proton secretion (Figure 7). For further experiments, 0.1 mM trehalose was chosen, as this was the only concentration significantly affecting proton secretion. For sulforaphane and 17β-estradiol, concentrations of 1 µM and 5 µM, respectively, were chosen, being in a similar concentration range to the synthetic agonists and antagonists.

### 2.8. ML-SI3 Did Not Reduce the Proton Secretion Induced by Histamine, Caffeine, Trehalose, and Sulforaphane

**Figure 8:**
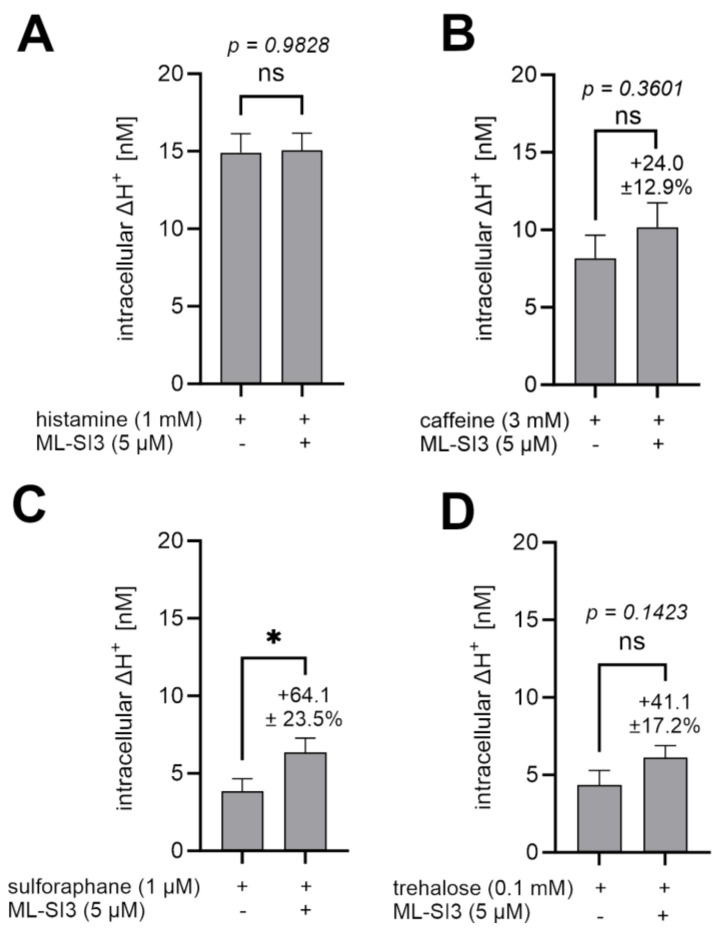
Effect of ML-SI3 on histamine-, caffeine-, trehalose-, and sulforaphane-induced proton secretion. Intracellular ΔH^+^ concentrations in nM are shown as mean ± SEM, *n* = 4–5, t. r. = 4–6. Statistics: Student’s *t*-test (two-tailed, unpaired); significant differences are indicated with ns = not significant; * = *p* ≤ 0.05. (**A**–**D**): Co-incubation of 5 µM ML-SI3 with 1 mM histamine, 3 mM caffeine, 1 µM sulforaphane, and 0.1 mM trehalose.

To verify the involvement of TRPML1 in the induction of proton secretion by sulforaphane and trehalose, the TRPML1 inhibitor ML-SI3 was applied. In addition, we also used ML-SI3 to analyze whether TRPML1 participates in two canonical pathways of proton secretion in HGT-1 cells: the H_2_-receptor-mediated cAMP-dependent pathway and the TAS2R-mediated Ca^2+^-dependent pathway. Therefore, 1 mM histamine and 3 mM caffeine, a well-described activator of proton secretion, at least via TAS2R7, 10, 14, 43, and 46 in HGT-1 cells [21,22,34], as well as 1 µM sulforaphane and 0.1 mM trehalose were used to analyze the impact of the pharmacological inhibition of TRPML1 upon applying the antagonist ML-SI3. We could not detect a reduction in proton secretion by co-incubating histamine with ML-SI3 (*p* > 0.05) (Figure 8A). Also, the induction of proton secretion by caffeine (*p* > 0.05), trehalose (*p* > 0.05), and sulforaphane was not reduced by the TRPML1 antagonist ML-SI3 (Figure 8B–D). In fact, a significant increase in proton secretion during co-incubation with ML-SI3 could be detected for sulforaphane (+64.1 ± 23.5%, *p* ≤ 0.05).

### 2.9. 17β-Estradiol Reduced the Proton Secretory Effect of ML-SA1 in HGT-1

**Figure 9:**
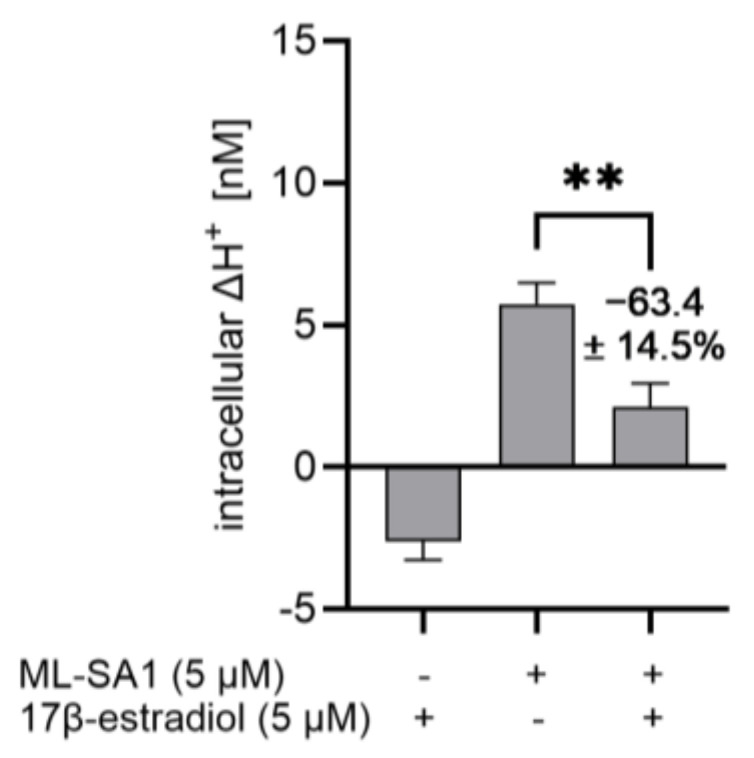
Inhibitory effect of 17β-estradiol on ML-SA1-stimulated proton secretion. Intracellular ΔH^+^ concentrations in nM are shown as mean ± SEM, *n* = 4–5, t. r. = 4–6. Statistics: Student’s *t*-test (two-tailed, unpaired); significant difference is indicated with ** = *p* ≤ 0.01.

Rühl et al. [16] showed that 17β-estradiol specifically inhibits the TRPML1 channel with an IC_50_ value of 5.3 µM after activation with 5 µM ML-SA1. Therefore, we decided to investigate the potential of 17β-estradiol (5 µM) to reduce the proton secretory effect of ML-SA1 (5 µM) on HGT-1 cells (Figure 9), thereby demonstrating a reduction in proton secretion by 63.4 ± 14.5% (*p* ≤ 0.01). This supports the findings of Rühl et al. [16] and indicates that 17β-estradiol in HGT-1 cells may influence proton secretion via the TRPML1 channel. 

### 2.10. Proton Secretion Induced by ML-SA1 and ML-SA5 Was Reduced in TRPML1 Knock-Down (kd) Cells 

**Figure 10:**
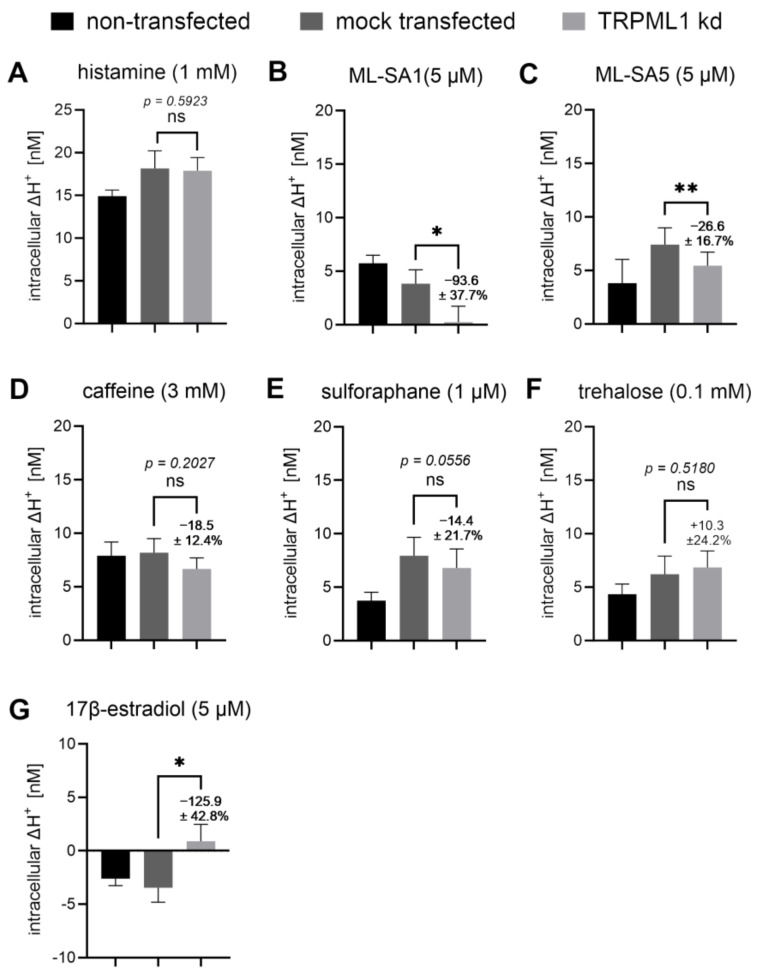
Histamine, caffeine, and TRPML1-targeting compounds and the influence on the proton-secreting effect in TRPML1 kd cells compared to mock-transfected and non-transfected HGT-1 cells. Intracellular ΔH^+^ concentrations in nM are shown for the comparison of non-transfected (black bar), mock-transfected (dark grey bar), and TRPML1 kd (light grey bar) cells incubated with 1 mM histamine (**A**), 5 µM ML-SA1 (**B**), 5 µM ML-SA5 (**C**), 3 mM caffeine (**D**), 1 µM sulforaphane (**E**), 0.1 mM trehalose (**F**), and 5 µM 17β-estradiol (**G**). Data are shown as mean ± SEM, *n* = 3–4, t. r. = 6; statistics: Student’s *t*-test (two-tailed, paired [mock vs. TRPML1 kd], unpaired [non-transfected vs. mock]); significant differences are indicated with ns = not significant; * = *p* ≤ 0.05; ** = *p* ≤ 0.01.

To further test our hypothesis that TRPML1 participates in proton secretion in HGT-1 cells, we performed a specific kd of TRPML1 in HGT-1 cells. The highest kd efficiency was determined via RT-qPCR after 72 h of transfection with 50 nM siRNA targeting TRPML1 (HSS126140). The expression of TRPML1 was reduced by 83.5 ± 3% (*p* ≤ 0.0001) at the mRNA level and 25.9% (*p* ≤ 0.0001) at the protein level (Figures S6 and S7). Mock-transfected cells showed no significant differences compared to non-transfected cells (Figure 10). The general involvement of TRPML1 in proton secretion via the kd approach was tested using the synthetic TRPML1 activators ML-SA1 and ML-SA5; the food-derived compounds caffeine, sulforaphane, and trehalose; and histamine (Figure 10). In this set of experiments, we could demonstrate a TRPML1-specific kd decrease in proton secretion compared to the mock-transfected cells for ML-SA1 (−93.6 ± 37.7%, *p* ≤ 0.05) and ML-SA5 (−26.6 ± 16.7% *p* ≤ 0.01) (Figure 10B,C). The effect of 17β-estradiol on ΔH^+^ concentrations was shifted toward the baseline in TRPML1 kd cells (−125.9 ± 42.8%, *p* ≤ 0.05). For sulforaphane, the respective decrease was borderline significant (−14.4 ± 21.7%, *p* = 0.0556). The specific kd of TRPML1 had no impact on proton secretion induced by histamine, caffeine, or trehalose (*p* > 0.05) (Figure 10A,D,F).

These results imply that TRPML1 is functionally involved in the induction of proton secretion by its activation in HGT-1 cells.

## 3. Discussion

In this study, we examined the TRPML1 channel in regard to its role in mechanisms regulating gastric acid secretion by using known agonists and antagonists as well as food-derived compounds that potentially target the TRPML1 channel in proton secretion assays and immunofluorescence staining to reveal its role in proton secretion and tubulovesicle trafficking in the human gastric parietal cell line HGT-1.

### 3.1. HGT-1 Cells Localize TRPML1 in or around the Cell Nucleus

First, we analyzed the protein expression and subcellular localization of TRPML1 in HGT-1 cells via immunocytochemistry. In addition to localization in the cytosol and at the cell membrane, TRPML1 was also detected in or around the nucleus. Other studies could not confirm the previously reported localization of TRPML1 channels in the nucleus of mouse parietal cells [7,19]. On the other hand, it was also found that TRPML1 is mainly expressed in the nucleus of PC-3, SK-MEL-30, U-2-OS, and glioblastoma cell lines [35,36,37], which may allow for the conclusion that the localization of TRPML1 in the nucleus could be characteristic of tumor cells. Furthermore, a nuclear localization motif (43–60 amino acids) was found in the amino acid sequence of the TRPML1 channel [18]. Sahoo et al. [7] detected the TRPML1 channel, mainly in Lamp-1-negative compartments in mouse parietal cells, compared with a high co-localization of 60–80% with the α- or β-subunit of H^+^/K^+^-ATPase. In contrast, in the work presented here, TRPML1 was found to have a relatively low mean co-localization of 9.1 ± 1.8% with the late endosome/lysosome marker Lamp-1 and the tubulovesicle marker H^+^/K^+^-ATPase (12.9 ± 0.7%) in HGT-1 cells. Also, the stimulation of the cells with histamine or ML-SA1 did not alter the co-localization rates of TRPML1 with Lamp-1 or H^+^/K^+^-ATPase. This presumably results from the strong signal of TRPML1 in the nucleus, where Lamp-1 and H^+^/K^+^-ATPase are not detectable. However, the co-localization of TRPML1 with Lamp-1 would also agree with the presence of a leucine zipper motif as a late endosomal/lysosomal targeting signal at the second transmembrane domain of the channel [18].

We could show that vacuolar apical compartment formation occurs in HGT-1 cells upon adding ML-SA1, indicating TV exocytosis due to the activation of the TRPML1 channel, which supported the hypothesis that TRPML1 activation is able to induce proton secretion.

### 3.2. ML-SA5 and ML-SA1 Impact Proton Secretion and Intracellular Calcium Concentrations via Different Mechanisms

The synthetic TRPML1 agonists ML-SA1 and ML-SA5 both stimulate proton secretion, indicating that activation of TRPML1 is sufficient for this process in HGT-1 cells. However, only the ML-SA1-induced proton secretion could be inhibited by the antagonist ML-SI3 (−122.2 ± 22.7%), while the ML-SA5-induced proton secretion was unaffected by the inhibitor. This could be explained by previous studies from Schmiege et al. [9], who described consistent binding sites for ML-SA1 and ML-SI3, namely the residues Phe465 in PH1, Leu422, Met426, Cys429, Val432, Ala433, and Tyr436 in S5 and Phe505 and Phe513 in S6, along with the residues Tyr499, Ser 503, Leu504, Tyr507, and Met508 in S6. Although the exact binding site(s) for ML-SA5 have not been described yet, it has been shown recently that this agonist is not selective for TRPML1, since ML-SA5 induced calcium mobilization with almost identical EC_50_ values in TRPML1-overexpressing cells compared to TRPML1 ko cells [17]. This aligns with our finding that the TRPML1 kd inhibited ML-SA1-induced proton secretion by about 94%, whereas the ML-SA5-induced proton secretion was reduced by about 27%. The fact that a TRPML1 kd with an efficiency of 25.9% at the protein level almost abolishes proton secretion induced by ML-SA1 may point to the requirement of cytosolic Ca^2+^ concentrations exceeding a threshold value to enable proton secretion. In isolated rat parietal cells, a decrease in basal Ca^2+^ by 12 ± 3% abolished the Ca^2+^ signal induced by 10 µM carbachol completely and inhibited the action of histamine by 70–100% [38]. This, however, would imply that TRPML1 may also participate in the regulation of basal Ca^2+^ levels, which would be supported by our finding that the TRPML1 antagonist 17β-estradiol affects the proton secretion of HGT-1 cells already in the resting state. However, this is speculative and needs to be clarified in future analysis.

Both ML-SA1 and ML-SA5 increased cytosolic Ca^2+^ concentrations, which were inhibited by the antagonist ML-SI3 completely for ML-SA1 and reduced by 35% for ML-SA5. This comparatively small reduction in the Ca^2+^ signal might be the reason for the finding that ML-SA5-induced proton secretion was not significantly diminished by ML-SI3. The absence of an increase in cytosolic Ca^2+^ concentrations upon the stimulation of HGT-1 cells with ML-SA1 and ML-SA5 in nominal Ca^2+^-free buffer indicates that the increase in cytosolic Ca^2+^ concentrations results from an influx of Ca^2+^ mediated by TRPML1 channels in the plasma membrane. This would be supported by the finding that the Ca^2+^ increase in HGT-1 cells is rather prolonged, whereas a release from intracellular stores would generate a transient signal, as was shown in TRPML1-overexpressing cells [7]. However, we cannot exclude an additional release of Ca^2+^ from acidic stores, which results in local Ca^2+^ increases that may not give rise to global Ca^2+^ increases.

### 3.3. 17β-Estradiol Reduced the Proton Secretory Effect of ML-SA1 on HGT-1 Cells

It is well established that food-derived substances can induce proton secretion in HGT-1 cells via their interaction with taste receptors [22,24]. Therefore, various food compounds triggering different signaling cascades were additionally tested with regard to (i) their potential to induce proton secretion and (ii) the involvement of TRPML1 in this process. First, sulforaphane, described as an agonist of TRPML1 [12], induced a mild but significant proton secretion by HGT-1 cells. Also, trehalose (0.1 mM) led to a secretion of protons from HGT-1 cells. For both substances, the intracellular ΔH^+^ concentrations after the stimulation of the cells reached about 4 nM. While sulforaphane was shown to directly interact with TRPML1 [12], trehalose was proposed to induce a transient lysosomal membrane permeability, causing Ca^2+^ release [14]. Whether this release is mediated via direct interaction with TRPML1 remains to be clarified. We cannot rule out the possibility that trehalose acts, at least partly, via TAS1R3, as shown by Ariyasu et al. [39], since the channel was expressed and functional in HGT-1 cells [28].

The described TRPML1 antagonist 17β-estradiol [16] decreased basal proton secretion at a concentration of 5 µM. Also, proton secretion induced by 5 µM ML-SA1 was reduced by 5 µM 17β-estradiol. This aligns with the results of Rühl et al. [16], who showed that 17β-estradiol has an inhibitory effect with an IC_50_ value of 5.3 µM on the TRPML1 channel. In contrast to ML-SI3, 17β-estradiol inhibits the TRPML1 channel with an IC_50_ of 4.7 µM after ML-SA1 activation [16]. A specific TRPML1 kd confirmed that 17β-estradiol indeed targets TRPML1. These results might indicate that in HGT-1 cells, TRPML1 affects basal proton secretion, which is in line with Chandra et al. [19], who showed that TRPML1 ko mice had significant impairments in basal gastric acid secretion. However, it is not clear whether food-derived 17β-estradiol could influence gastric acid secretion since the respective concentrations in foods are known to be low, ranging from 0.02 µg/L (0.073 nM) in cow milk to 1.0 ± 0.2 pg/mg egg yolk [40,41].

### 3.4. Histamine and the Food-Derived Compounds Trehalose and Caffeine Do Not Act via TRPML1 in HGT-1 Cells

Regarding the canonical pathways, it is known that histamine induces gastric acid secretion via the activation of H_2_ receptors and subsequent cAMP-mediated PKA activation [42,43]. The activation of TAS2Rs by bitter compounds such as caffeine leads to a dissociation of the G protein G_αgustducin_ from the β3γ13 subunit, thereby activating the phospholipase Cβ2 (PLCβ2) to cleave phosphatidylinositol-4,5-bisphosphate (PtdIns (4,5) P_2_) into inositol-1,4,5-trisphosphate (IP_3_) and diacylglycerol (DAG) [22,43]. IP_3_ then activates the IP_3_ receptor, mobilizing intracellular Ca^2+^ from the ER to the cytosol, resulting in gastric acid secretion [22,43]. This pathway shares common features with the Ca^2+^-dependent gastric acid secretion induced by acetylcholine via the muscarinic M_3_ receptor [42]. Also, interactions between the cAMP- and Ca^2+^-dependent pathways may occur [38]. However, it is still poorly understood how the cAMP and Ca^2+^ signals initiate proton secretion by the H^+^/K^+^-ATPase. Sahoo et al. [7] showed for histamine that TRPML1 induces a cAMP-mediated, PKA-sensitive Ca^2+^ release from TVs, promoting a Ca^2+^-dependent fusion of TVs containing the H^+^/K^+^-ATPase with each other and the apical membrane for acid secretion in mice. This consequently implies that the cAMP production and the subsequent Ca^2+^-release from TVs are essential for proton secretion.

To clarify whether TRPML1 participates in parietal proton secretion via the cAMP- and/or the Ca^2+^-dependent pathway, we first applied the TRPML1 antagonist ML-SI3 to pharmacologically inhibit TRPML1. ML-SI3, however, did not reduce the proton secretion induced by the tested food-derived compounds and histamine. For sulforaphane, even an increased proton secretion during co-incubation with ML-SI3 was detected. Only ML-SA1-activated TRPML1 could be completely inhibited by ML-SI3 in HGT-1 cells. This may be because ML-SI3 can only interfere with compounds binding to the same binding cavity, as it cannot alter the activation by PI(3,5)P_2_, addressing a different binding site [9].

Using a TRPML1-specific kd, we could demonstrate the participation of TRPML1 in proton secretion for ML-SA1 and partly for ML-SA5. The kd of TRPML1, however, had no effect on histamine-induced proton secretion. If histamine application actually induces increases in cytosolic Ca^2+^ via TRPML1 in HGT-1 cells, these increases could be considered to occur via TRPM4/M5, which was not triggered upon histamine application [21]. This is in contrast to the results in mice [7] but in line with the finding that Ca^2+^ signaling from lysosomes is not impaired in TRPML1 ko mice [19]. Therefore, it remains unclear whether Ca^2+^ efflux from lysosomes or TVs generally is the key mechanism behind the TRPML1 signaling cascade.

One explanation, however, could be that this mechanism is species specific, presumably due to species-dependent engagement of different G_α_ proteins. In canine parietal cells, H_2_ receptor downstream signaling does not cause an increase in intracellular Ca^2+^ [44]. Contrarily, it could be demonstrated that histamine-induced H_2_ receptor activation can lead to both Ca^2+^-dependent and -independent signaling pathways in rabbit parietal cells [45]. In isolated rat parietal cells, cAMP- and Ca^2+^-mediated signaling are necessary for proton secretion [38]. In human parietal cells, it could be both or even just a Ca^2+^-independent pathway. Also, the TRPML1 channel differs between species. A sequence alignment using Clustal W showed a higher score for the human TRPML1 channel (UniprotID: Q9GZU1) with the rabbit TRPML1 channel (UniprotID: G1T1X3), at 94.3, than for the murine TRPML1 channel (UniprotID: Q99J21), at 91.3, and for known rat TRPML1 sequences (UniprotIDs: D3ZRF9, A0A8I6AQ69, A0A8I6G3L4), with scores between 91.0 and 91.4 [46,47,48].

However, proton secretion via Ca^2+^-dependent TAS2R stimulation by caffeine does not seem to be reliant on TRPML1 channels, at least on the basis of the results presented here. Proton release stimulated by 3 mM caffeine was neither inhibited by 5 µM ML-SI3 nor reduced in TRPML1 kd cells. Consequently, this also suggests that proton secretion via TAS2Rs appears to be completely independent of TRPML1 signaling in HGT-1 cells, at least that triggered by caffeine. This might indicate that Ca^2+^ is necessary for proton secretion in HGT-1 cells, but the process does not necessarily depend on Ca^2+^ release from TVs via TRPML1. 

On the other hand, TRPML1 might be involved in the induction of proton secretion by sulforaphane, which tended to be decreased in TRPML1 kd cells. The incubation of sulforaphane with the TRPML1 antagonist ML-SI3 did not show a reduction in proton secretion, indicating that sulforaphane’s activation mechanism is different from ML-SA1-mediated TRPML1 activation. Sulforaphane appears to be an interesting food-related compound involved in TRPML1-regulated gastric acid secretion, independent of direct TAS2R activation. However, since the structurally related allylisothiocyanyte is known to be bitter and activates TAS2R38 [34], it also can not be ruled out that other signaling pathways might be involved. Whether sulforaphane can affect gastric acid secretion via the TRPML1-mediated release of Ca^2+^ ions after the consumption of sulforaphane-containing foods remains to be elucidated. 

## 4. Material and Methods

### 4.1. Chemicals 

Chemicals used in assays were dissolved and stored at −20 °C. ML-SA1 (purity ≥ 98%) was bought from Bio-Techne, Wiesbaden, Germany; ML-SA5 (purity ≥ 98%) from Merck KGaA, Darmstadt, Germany; and ML-SI3 (purity ≥ 98%) from BIOZOL Diagnostics Vertrieb GmbH, Eching, Germany, and dissolved in DMSO. D-(+)-trehalose dehydrate (purity ≥ 99%) dissolved in ddH_2_O and 17β-estradiol (purity ≥ 98%) in EtOH were bought from Merck KGaA, Germany. Sulforaphane (purity ≥ 98.19%) dissolved in DMSO, 1× DMEM Glutamax, Fluoromount-G^TM^, 16% formaldehyde solution methanol-free, MTT (3-(4,5-dimethylthiazol-2-yl)-2,5-diphenyltetrazolium bromide), siRNAs HSS126140 and HSS126141, Lipofectamine RNAiMAX, Opti-Medium, and SNARF-1-AM were purchased from Fisher Scientific GmbH, Munich, Germany. FBS and penicillin–streptomycin (10,000 U/mL penicillin, 10 mg/mL streptomycin) were bought from PAN-Biotech GmbH, Aidenbach, Germany, and horse serum from Biowest, Nuaillé, France.

### 4.2. Cell Culture

The human gastric parietal cell line HGT-1 (RRID: CVCL_A609) was provided by C. Laboisse (Laboratory of Pathological Anatomy, Nantes, France). HGT-1 cells were cultured in cell culture flasks at 37 °C and 5% CO_2_ (standard conditions) in 1× DMEM (10% FBS and 1% penicillin–streptomycin). Experiments were performed with cells between passages 15 and 30.

### 4.3. Immunostaining and Confocal Laser Scanning Microscopy (CLSM)

For immunostaining, 25,000–50,000 HGT-1 cells were seeded at standard conditions for 24 h in a poly-D-lysine (10 μg/mL)-coated glass-bottom 10-well plate (GreinerBio-One GmbH, Frickenhausen, Germany). If the influence of different compounds was to be investigated, the cells were washed with 37 °C prewarmed PBS buffer and treated with the substances diluted in PBS buffer for 10 min at standard conditions. The immunostaining of HGT-1 cells was then performed as described in Richter and Andersen et al. [21]. If the actin filaments were stained, the cells were incubated with DyLight ™ 554 Phalloidin (Cell Signaling Technology, Danvers, MA, USA) nucleus stain. The used stains, primary and secondary antibodies, and dilutions are shown in Table 1.

Images were acquired using a Zeiss LSM 780 microscope (Carl Zeiss AG, Munich, Germany) with a 40×/1.2 Imm Korr DIC M27 objective lens, LSM T-PMT detector, and the ZEN 2.3 SP1 black program. An airyscan detector was used to obtain pictures with high resolution. The ZEN 2.3 SP1 blue program was used to detect co-localization. Co-localization was then calculated by dividing the pixels that detected both fluorescence signals by the total pixels counted with the signal minus the background pixels.

### 4.4. Cell Viability 

To measure cell viability, 100,000 cells were seeded in a transparent 96-well plate and incubated overnight. Before treating the cells with the compounds of interest for 30 min at standard conditions, the cells were washed with KRHB (10 mM HEPES, 11.7 mM D-glucose, 4.7 mM KCl, 130 mM NaCl, 1.3 mM CaCl_2_, 1.2 mM MgSO_4_, and 1.2 mM KH_2_PO_4_, pH 7.4). Then, 0.83 mg/mL MTT in DMEM was incubated for 15 min under standard conditions. The formed formazan was dissolved in DMSO and measured at 570 nm (reference wavelength 650 nm) in an Infinite M200 plate reader (Tecan, Männedorf, Switzerland). Pure 100% DMSO served as a negative control, and KRHB-only treated cells (=100%) were used for normalization to calculate cell viability. For 10 µM sulforaphane, 500 µM trehalose, 100 µM 17β-estradiol, 20 µM ML-SA, 10 µM ML-SA5, and 10 µM ML-SI3, cell viability was not affected (*p* > 0.05) in HGT-1 cells (Figure S8).

### 4.5. Proton Secretion Assay

To determine the intracellular pH alteration in HGT-1 cells after incubating with different effectors, an assay for proton secretion detection with the pH-sensitive fluorescent dye SNARF-1-AM was used as developed and applied previously [20,22,23,29]. For this, 100,000 viable cells were seeded per well in a black 96-well plate and incubated for 20–24 h. On the day of the experiment, the cells were washed with KRHB and stained with 3 µM SNARF-1-AM for 30 min under standard conditions. After a washing step with KRHB, the cells were incubated with the test substances in the desired concentrations. In each assay, 1 mM of histamine served as a positive control. The proportion of solvents DMSO and ethanol were max. 0.1% in each probe. The pH was calculated by applying a calibration curve with various pH values in the presence of 20 µM nigericin. After 12 min incubation, fluorescence was detected at 580 and 640 nm emission after excitation at 488 nm using the plate reader FlexStation 3 (Molecular Devices, San Jose, CA, USA). After determining intracellular H^+^ concentrations from the intracellular pH values, changes in the intracellular H^+^ concentration (ΔH^+^) were calculated by subtracting treated cells from untreated cells. The ΔH^+^ describes how many protons were secreted compared to untreated cells. To compare values from different experiments, the data were normalized to the positive control with 1 mM histamine.

### 4.6. Transient Knock-Down of TRPML1

The protein expression of TRPML1 was reduced by the degradation of TRPML1 mRNA through small interfering RNA (siRNA). Transfection efficiency was measured after transfection was performed according to the manufacturer’s protocol and as already published for HGT-1 cells [20]. Briefly, Lipofectamine RNAiMAX in Opti-Medium was mixed with the siRNA in Opti-Medium at final concentrations of 1 nM, 10 nm, and 50 nM with either of the two siRNAs, HSS126140 and HSS126141. A 10 nM concentration of mock siRNA as a negative control and 10 nM MAPK1 (VHS40312) as a positive control were used analogously. The transfection rate was determined by RT-qPCR after RNA isolation. For the proton secretion assay, 20,000 cells per well were seeded 24 h before transfection with 50 nM siRNA (TRPML1: HSS126140) into a black 96-well plate. Mock transfection as a negative control was performed analogously with 50 nM unspecific siRNA. After 72 h of transient transfection, the proton secretion assay was performed with TRPML1 knock-down HGT-1 cells and mock-transfected HGT-1 cells for comparison.

### 4.7. Relative Quantitation of RNA with qPCR and Real-Time PCR 

First, RNA from HGT-1 cells was isolated using the peqGOLD MicroSpin Total RNA Kit (VWR International GmbH, Ismaning, Germany) according to the manufacturer’s instructions. Then, DNase digestion and cDNA synthesis were performed using an iSCriptTM gDNA Clear cDNA Synthesis Kit (Bio-Rad Laboratories GmbH, Feldkirchen, Germany) according to the manufacturer’s instructions in a C1000 Touch Thermal Cycler (Bio-Rad Laboratories GmbH, Feldkirchen Germany). RT-qPCR was performed with 50 ng of cDNA and the Advanced Universal SYBR^®^ Green Supermix (Bio-Rad Laboratories GmbH, Feldkirchen Germany) in a C1000 Touch Thermal Cycler (Bio-Rad Laboratories GmbH, Feldkirchen Germany) equipped with a CFX96TM Real-Time System (Bio-Rad Laboratories GmbH, Feldkirchen Germany). The 500 nM primers for TRPML1 were used as previously published [49]. The primers for MAPK1 and the two reference genes, peptidylprolyl isomerase A (PPIA [29]) and glyceraldehyde-3-phosphate dehydrogenase (GAPDH [50]), are listed in Table S1; assay controls were purchased from Bio-Rad Laboratories GmbH, Feldkirchen Germany. The difference between the Ct mean of the gene of interest and that of the GAPDH and PPIA was calculated for the relative quantitation of gene expression.

### 4.8. Flow Cytometry 

To investigate the tubulovesicle and lysosomal fusion with the cell membrane upon stimulation, flow cytometry experiments were performed with an antibody against Lamp-1. For this purpose, 100,000 cells in 1×DMEM without phenolic red were incubated with different compounds for 10 min. Then, 0.25 µg of CD107a (LAMP-1) antibody, PE-Cyanine5 (Fisher Scientific GmbH, Germany, RRID: AB_10547280), per 100 µL cell suspension was added and incubated again for 5 min at RT. Before the cells were applied to the flow cytometer MACSQuant^®^ Analyzer 16 (Miltenyi Biotec B.V.&Co. KG, Bergisch Gladbach, Germany), each sample was diluted with 500 µL of PBS buffer. Data were analyzed using the FlowLogic^TM^ (Inivai Technologies, Mentone, Australia) program.

### 4.9. Intracellular Ca^2+^ Mobilization Assay

Intracellular Ca^2+^ mobilization was measured as described by Richter and Andersen et al. [21]. Briefly, 50,000 cells per well in a black 96-well plate with a transparent bottom were grown for 20–24 h. After one washing step with KRHB, the cells were incubated with 1 µM Cal-520 AM (in KRHB with 0.02% DMSO and 0.004% Pluronic F-127) for 2 h under standard conditions. Measurements (excitation: 495 nm, emission: 515 nm) were performed in 2 s intervals in a FLIPR^TETRA^ system (Molecular Devices, San Jose, CA, USA). After 60 s, the compounds of interest were applied automatically, and the fluorescence was recorded continuously. Relative fluorescence (RFU) was calculated relative to baseline fluorescence. For experiments without Ca^2+^, KRHB without CaCl_2_ and with 1 mM EGTA was added to the cells before fluorescence measurement.

### 4.10. Statistical Analysis

Data were analyzed using GraphPad Prism 9.4.0. Values are presented as mean ± standard error of the mean (SEM) if not mentioned otherwise. Data from the proton secretion assay were subjected to a Nalimov outlier test and analyzed with a two-tailed Student’s *t*-test of untreated cells compared to treated cells, if not mentioned otherwise. Depending on whether the values were compared in one assay with the same biological or technical replicates, paired values were analyzed; otherwise, they were unpaired. The test used is indicated in the respective figure legends. Different *p* values are indicated with asterisks according to the following scheme: * = *p* ≤ 0.05, ** = *p* ≤ 0.01, *** = *p* ≤ 0.001, **** = *p* ≤ 0.0001.

## 5. Conclusions

This study provides first evidence that the direct activation of the Ca^2+^ channel TRPML1 can induce proton secretion in the human gastric parietal cell line HGT-1. We could demonstrate that the TRPML1 agonists ML-SA1 and ML-SA5 triggered proton secretion in HGT-1 cells that was reduced in TRPML1 kd cells. Furthermore, the TRPML1 inhibitor 17β-estradiol inhibited proton secretion in resting HGT-1 cells as well as upon the stimulation of the cells with ML-SA1. On the other hand, TRPML1 does not seem to participate in histamine- and caffeine-induced proton secretion. These results suggest that TRPML1 can play a role in proton secretion in HGT-1 cells, but (i) this role depends on the activation or inhibition of the channel itself, (ii) it is insufficient for full induction, and (iii) TRPML1 is not involved in canonical signaling cascades, especially in histamine-stimulated proton secretion. It seems feasible that the TRPML1 channel can regulate proton secretion, but it very likely works in conjunction with other cellular mechanisms. It will be of great interest to identify the endogenous signals leading to proton secretion via TRPML1.

## Figures and Tables

**Table 1:** Stains and antibodies.

Staining Antibody/Chemical	Company	Working Solution
Hoechst-33342	Fisher Scientific GmbH, Munich, Germany	1:2000
Concanavalin A biotin conjugate	Sigma-Aldrich, Munich, Germany	1:2000
Streptavidin, Alexa Fluor™ 633 conjugate (RRID:AB_2313500)	Fisher Scientific GmbH, Munich, Germany	1 µg/mL
DyLight^™^ 554 Phalloidin	Cell Signaling Technology, Beverly, MA USA	1:100
Anti-TRPML1 antibody (RRID: AB_10915894)	Alomone labs, Jerusalem, Israel	8 µg/mL
TRPML1 blocking peptide	Alomone labs, Jerusalem, Israel	8 µg/mL
Alexa Fluor 488 anti-Rabbit IgG (RRID: AB_2536097)	Fisher Scientific GmbH, Munich, Germany	1 µg/mL
Anti-human-CD107a (LAMP-1) (RRID:AB_467426)	Fisher Scientific GmbH, Munich, Germany	10 µg/mL
Anti-ATP4B Monoclonal Antibody (2G11) (RRID:AB_2227726)	Fisher Scientific GmbH, Munich, Germany	1:100
eFluor^™^ 570 anti-Mouse IgG (RRID: AB_2573606)	Fisher Scientific GmbH, Munich, Germany	1 µg/mL

## Data Availability

The data generated during and/or analyzed during the current study are not publicly available due to the institutional statutes but are available from the corresponding author on reasonable request.

## Supplementary Materials

The following supporting information can be downloaded at: https://www.mdpi.com/article/10.3390/ijms25168829/s1.
