# Supplementary material for: Activation of the TRPML1 Ion Channel Induces Proton Secretion in the Human Gastric Parietal Cell Line HGT-1
Source: Int J Mol Sci. 2024 Aug 13;25(16):8829. doi: 10.3390/ijms25168829 (PMC11354443; doi:10.3390/ijms25168829)
Supplement: Supplementary file 1 [file ijms-25-08829-s001.zip › ijms-3099439-supplementary.pdf]

Supplementary Materials

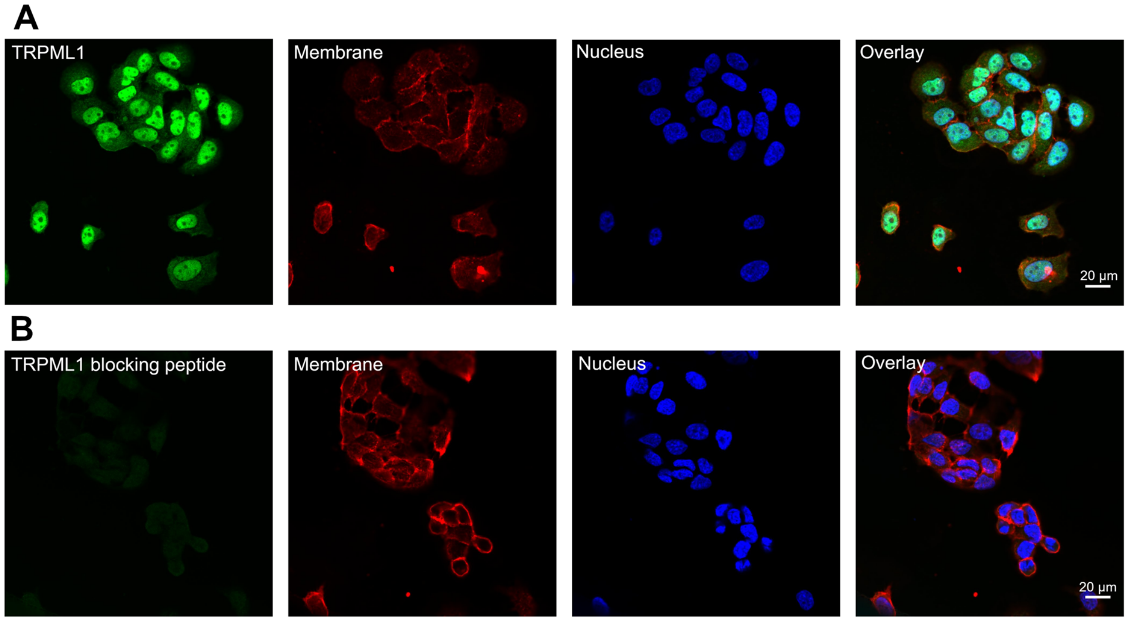

Figure S1. Detection of TRPML1 channels in HGT-1 cells by immunocytochemistry: TRPML1 channels in HGT-1 cells were stained with anti-TRPML1 antibody and secondary antibody anti-Rabbit IgG with Alexa Fluor 488 fluorophore, depicted in green. ConA and the secondary antibody streptavidin-Alexa Fluor 633 were chosen for cell membrane staining, shown in red. The fluorophore Hoechst-33342 was used to stain the nucleus, shown in blue. The Zeiss LSM 780 microscope and a 40x/1.2 Imm Korr DIC M27 objective lens were used for image acquisition. Scale bar represents 20 µm. A: Fluorescence labeling of TRPML1 in HGT-1 cells with anti-TRPML1 antibody. B: Anti-TRPML1 antibody specificity control with TRPML1 blocking peptide applied 1:1 with the anti-TRPML1 antibody.

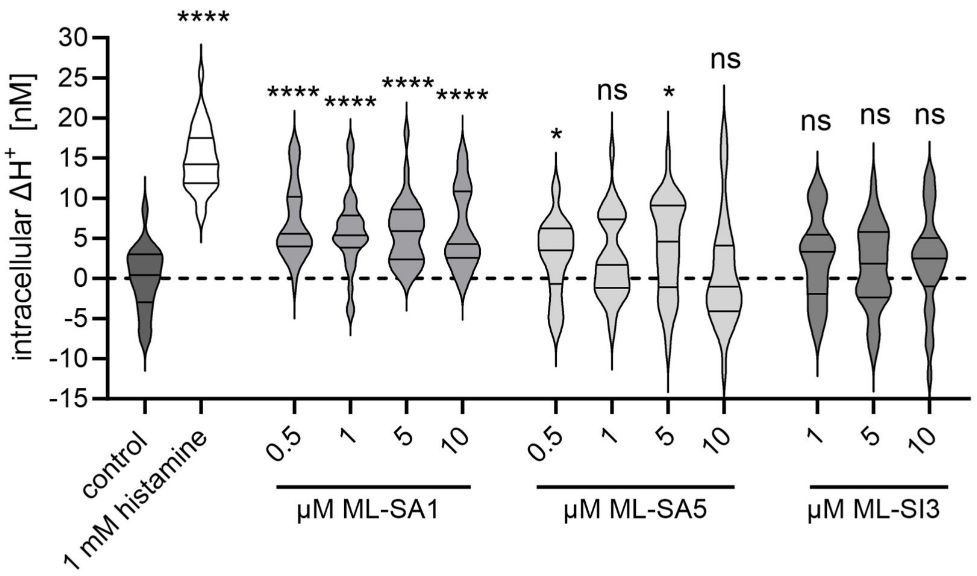

Figure S2. Effect on proton secretion of HGT-1 cells incubated with synthetic molecules ML-SA1, ML-SA5, and ML-SI3: To determine the intracellular pH alteration in HGT-1 after incubating with agonists ML-SA1 or ML-SA5 and antagonist ML-SI3, an assay for proton secretion detection was used. A concentration of 1 mM histamine serves as positive control. Intracellular ΔH^+ concentrations in nM are shown as violin plot, n = 4-5, t. r. = 4-6. Statistics: Student's t-test (two-tailed, unpaired); significant differences are expressed with * = $p \leq 0.05$; **** = $p \leq 0.0001$.

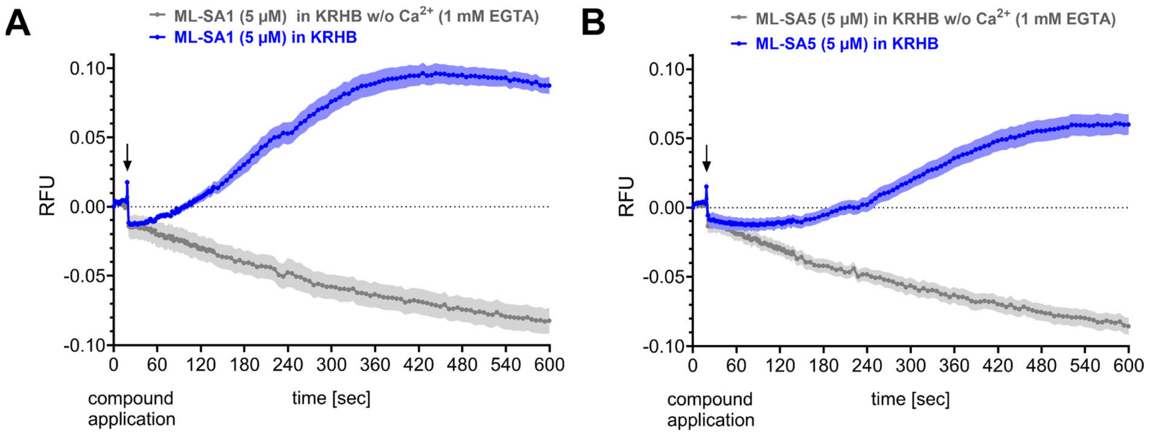

Figure S3. Relative fluorescence signal of Ca^{2+} triggered by 5 µM ML-SA1 (**A**) and 5 µM ML-SA5 (**B**) in KRHB with or without Ca^{2+} and 1 mM EGTA measured with the calcium mobilization assay in HGT-1 cells. Data is normalized to baseline fluorescence n = 3, t. r. = 3.

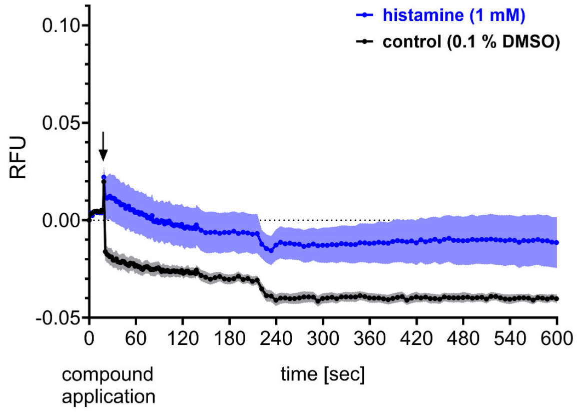

Figure S4. Relative fluorescence signal of Ca^{2+} triggered by 1 mM histamine in HGT-1 cells in calcium mobilization assay. Data is normalized to baseline fluorescence n = 3, t. r. = 3.

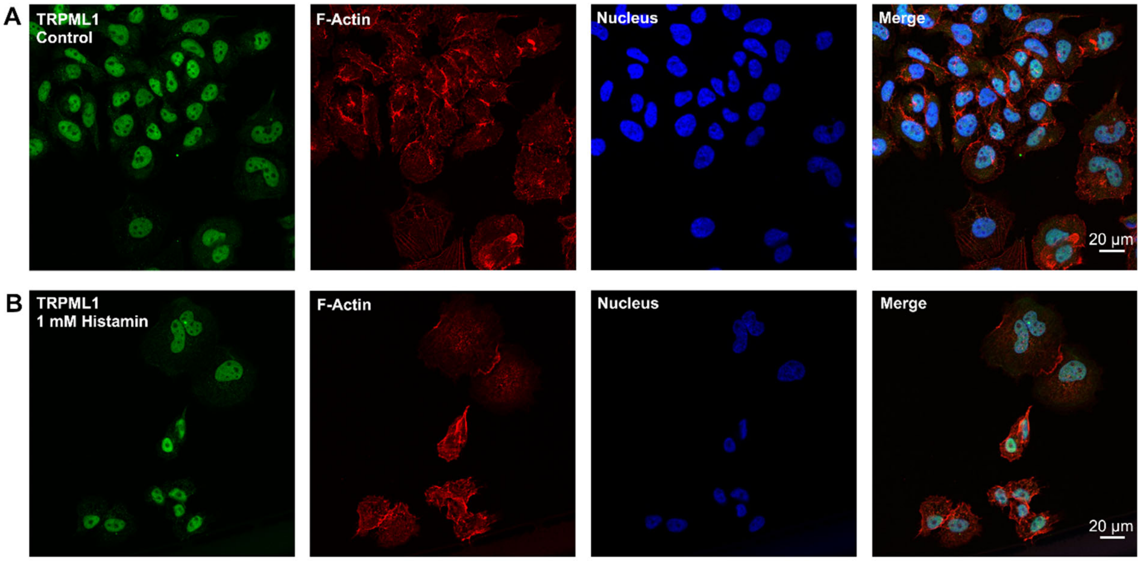

Figure S5. TRPML1 and actin staining in HGT-1 cells. To detect the TRPML1 channel in HGT-1 cells, the cells were incubated with anti-TRPML1 antibody and secondary antibody anti-Rabbit IgG with Alexa Fluor 488 fluorophore, depicted in green. DyLight™ 554 Phalloidin was chosen for F-actin staining, shown in red. The fluorophore Hoechst-33342 was used to stain the nucleus, shown in blue. The cells were preincubated with 1 mM histamine in PBS for 10 min before staining. The Zeiss LSM 780 microscope and a 40x/1.2 Imm Korr DIC M27 objective lens were used for image acquisition. Scale bar represents 20 µm.

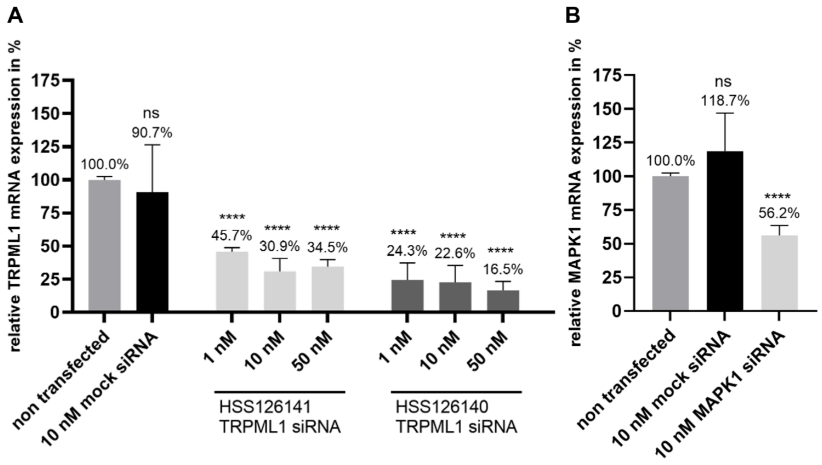

Figure S6. Relative mRNA expression after transient transfection (knock-down) with siRNA: HGT-1 cells were transfected with Lipofectamine with different siRNA in different concentrations to determine the best condition after 72 h. Mock transfections were performed with siRNA without target serving as negative control. MAPK1 knock-down with specific targeting siRNA was used as positive control. Relative mRNA expression was determined after RNA isolation and RT-qPCR. Values were normalized to non-transfected cells set to 100%. Reduction in expression was best achieved with 50 nM siRNA targeting TRPML1 (HSS126140) to 16.5%. Data are shown as geometric mean ± 95% CI, n = 4, t. r. = 3. Statistics: one-way ANOVA (two-tailed, unpaired) significant differences are expressed with **** = p ≤ 0.0001.

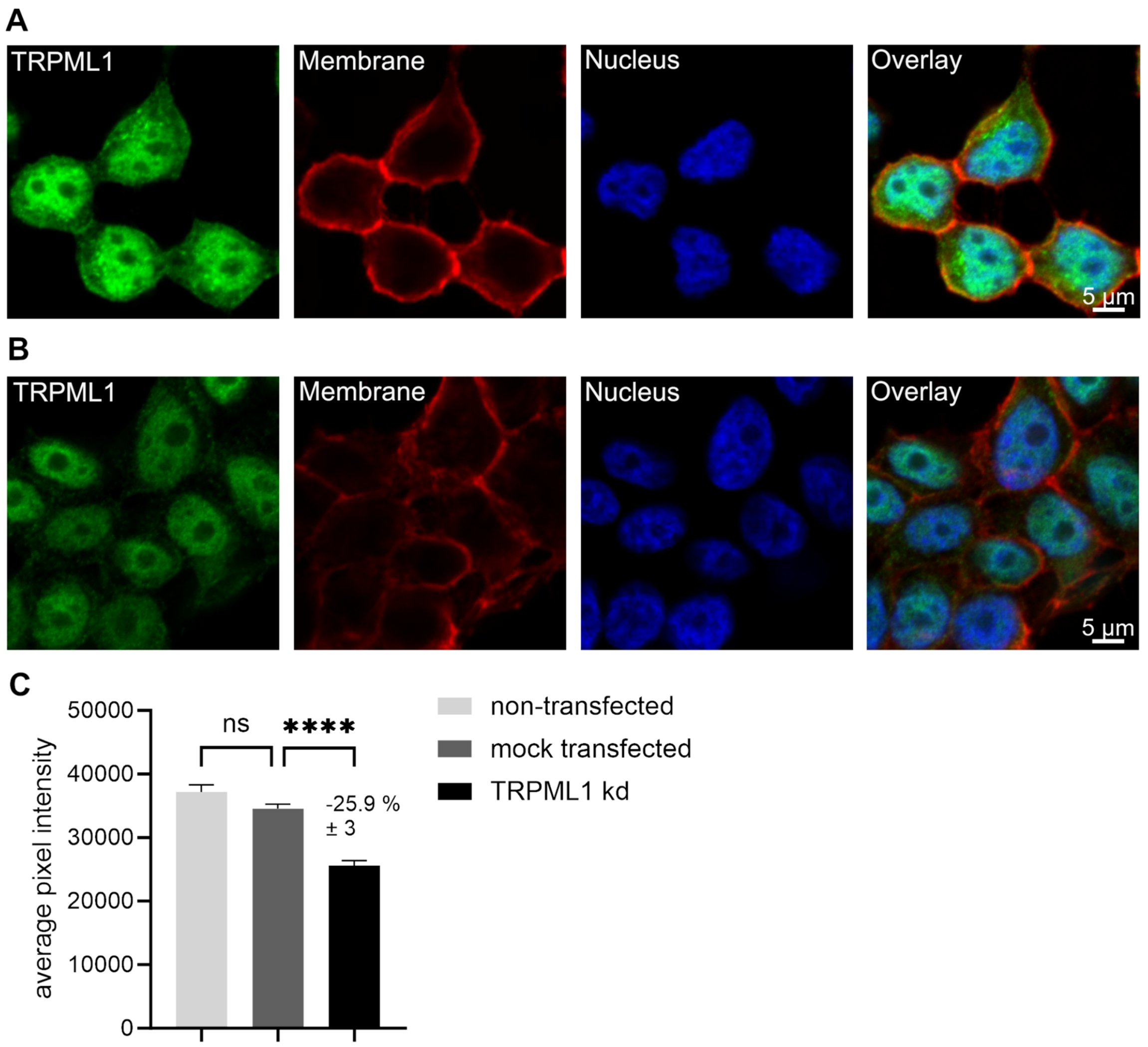

Figure S7. Immunocytochemistry of TRPML1 siRNA and mock-transfected HGT-1 cells to detect reduced TRPML1 expression: HGT-1 cells were transfected with either 50 nM TRPML1 siRNA or 50 nM mock as negative control compared to non-transfected cells. After 72 h, the TRPML1 channels in HGT-1 cells were stained with anti-TRPML1 antibody and secondary antibody anti-Rabbit IgG with Alexa Fluor 488 fluorophore depicted in green. ConA and the secondary antibody streptavidin-Alexa Fluor 633 were chosen for cell membrane staining, shown in red. The fluorophore Hoechst-33342 was used to stain the nucleus, shown in blue. Scale bar represents 5 µm. In A, the mock-transfected cells are depicted. In B, the TRPML1 knock-down cells are shown. In C, the average pixel intensity for the measured cells is shown.

25 cells were analyzed for each condition with ImageJ 1.50b. Data are shown as mean ± SEM, n = 25, t. r. = 3. Statistics: Student's t-test (two-tailed, paired); ns: not significant.; significant differences are expressed with: **** = p ≤ 0.0001.

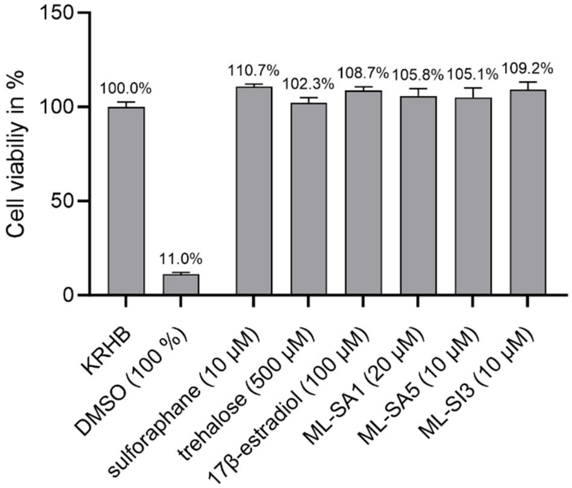

Figure S8. Cell viability assay of HGT-1 cells with sulforaphane, trehalose, and 17β-estradiol, ML-SA1, ML-SA5 and ML-SI3: The impact of substances on cell viability was measured using the tetrazole MTT (3-(4,5-dimethylthiazol-2-yl)-2,5-diphenyltetrazolium bromide) reagent. Compounds of interest in the desired concentrations were incubated for 30 min. DMSO, and KRHB were used as controls. To determine the cell viability, the absorption was measured at 570 nm and calculated relative to the KRHB-only treated control cells. Data was normalized to KRHB treated cells set to 100% and are shown as mean ± SEM, n = 3, t. r. = 3.

Table S1. RT-qPCR primer pairs

Gene	Direction	Sequence (5′ to 3′)
TRPML1	*forward*	CAG CGT CCT GAT CAC GTT TG
	reverse	GCT GTT GTC TCC GTG CTG G
PPIA	*forward*	CCA CCA GAT CAT TCC TTC TGT AGC
	reverse	CTG CAA TCC AGC TAG GCA TGG
GAPDH	*forward*	AGG TCG GAG TCA ACG GAT TTG
	reverse	GGG GTC ATT GAT GGC AAC AAT A

For MAPK1, verified primers (qHsaCEP0050000) were obtained (Bio-Rad Laboratories GmbH, Germany)
